# Endometriosis and Adenomyosis: Modern Concepts of Their Clinical Outcomes, Treatment, and Management

**DOI:** 10.3390/jcm13143996

**Published:** 2024-07-09

**Authors:** Jessica Ottolina, Roberta Villanacci, Sara D’Alessandro, Xuemin He, Giorgia Grisafi, Stefano Maria Ferrari, Massimo Candiani

**Affiliations:** 1 IRCCS San Raffaele Scientific Institute, Via Olgettina 60, 20132 Milan, Italy; villanacci.roberta@hsr.it (R.V.); dalessandro.sara@hsr.it (S.D.); grisafi.giorgia@hsr.it (G.G.); ferrari.stefano@hsr.it (S.M.F.); candiani.massimo@hsr.it (M.C.); 2 Obstetrics and Gynecology Unit, Department of Biomedical Sciences and Human Oncology, University of “Aldo Moro”, 70124 Bari, Italy; he.xuemin@hsr.it

**Keywords:** endometriosis, superficial peritoneal endometriosis, ovarian endometriosis, deep infiltrating endometriosis, adenomyosis

## Abstract

Endometriosis and adenomyosis are complex gynecological conditions characterized by diverse clinical presentations, including superficial peritoneal endometriosis (SPE), ovarian endometrioma (OMA), and deep infiltrating endometriosis (DIE). The hallmark features of these pathologies involve the manifestation of pain symptoms and infertility, and approximately 30% of patients are asymptomatic. Despite ongoing research, definitive treatments for these conditions remain elusive, and clinical management primarily revolves around medical or surgical interventions. Recent advancements in our understanding of the efficacy of various treatment modalities, including medical therapy and surgical interventions, have provided clinicians with valuable insights into pain relief and fertility preservation. This review aims to provide an updated overview of the latest literature on clinical outcomes, treatment options, and management strategies for different types of endometriosis. By synthesizing the newest available data, this review seeks to inform clinicians and guide decision making based on factors such as patients’ symptom severity, childbearing desire, and overall health.

## 1. Introduction

Endometriosis is a chronic inflammatory disease that significantly impacts a patient’s quality of life, social interactions, and work. It is primarily characterized by pain, often associated with the menstrual cycle, and infertility. The exact prevalence of endometriosis is unknown but estimates range from 2% to 10% of the general female population and up to 70% of infertile women [1,2]. Endometriosis is often categorized as peritoneal endometriosis, deep infiltrating endometriosis (DIE), or ovarian endometriosis based on the type and location of lesions. Adenomyosis is another related condition characterized by the infiltration of endometrial glands and stroma into the myometrium, leading to the hypertrophy of adjacent myometrial tissue.

Definitive treatment for endometriosis and adenomyosis is lacking, and management typically involves medical or surgical interventions. In certain cases, international and national guidelines agree on first-line treatment options, but in other cases, there remains no consensus within the scientific community.

New evidence from recent studies has helped improve our understanding and treatment of endometriosis-related pain and infertility [3]. This narrative review aims to provide an overview of the latest data, analyzing recent guidelines, randomized controlled trials (RCTs), cohort studies, and meta-analyses on the clinical outcomes, treatment, and management of different types of endometriosis.

## 2. Materials and Methods

A comprehensive literature search was conducted in PubMed, covering the period from January 2000 to December 2023. The research utilized combinations of the search terms “ovarian endometriosis”, “superficial peritoneal endometriosis”, “deep infiltrating endometriosis”, and “adenomyosis”, in conjunction with “infertility”, “pain”, and “quality of life”.

The inclusion criteria were limited to publications in English. The selection criteria encompassed all published randomized controlled trials (RCTs) and non-randomized studies (NRSs), including observational studies, prospective and retrospective cohort studies, and case-control studies, specifically focusing on the treatment of pain and infertility in endometriosis. Both original research articles and review articles were considered.

**Figure 1:**
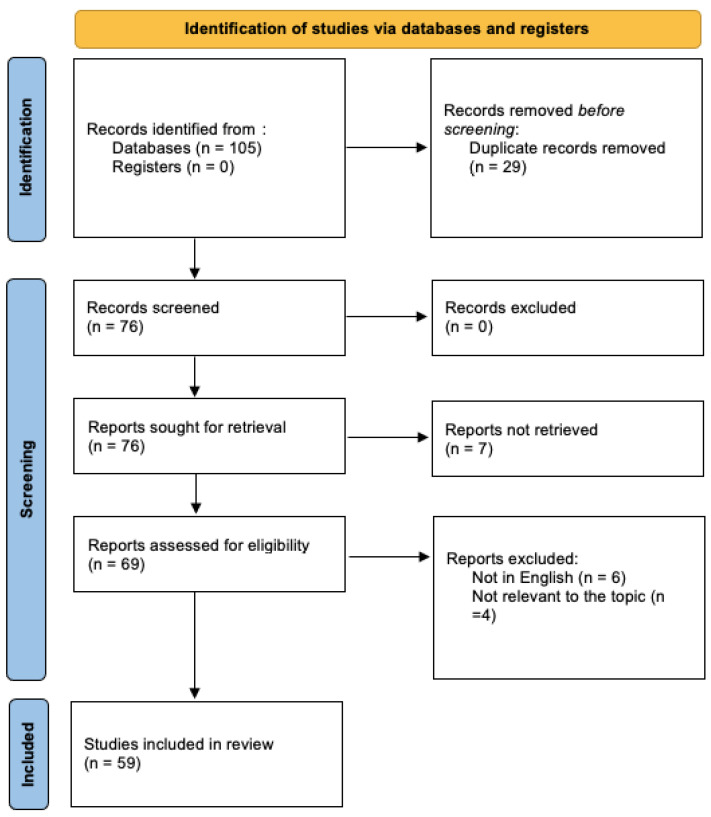
PRISMA flow diagram of the article selection process.

Additional relevant articles were identified by examining the references of the retrieved reviews and utilizing PubMed’s “similar articles” function for all of the selected records. After removing duplicates and articles that were not pertinent to the topic, a total of 59 papers were identified for inclusion in this narrative review (Figure 1).

## 3. Results

### 3.1. Endometriosis

#### 3.1.1. Ovarian Endometriosis

Ovarian endometriomas or “chocolate cysts” are ovarian cysts filled with menstrual blood. The endometrioma wall usually comprises a flattened columnar epithelium with an endometrial-type stroma, surrounded by fibroreactive tissue with haemosiderin-laden macrophages. However, histologic analyses have shown that endometrial-like tissue often is minimal or absent (especially in larger cysts), and only fibrous tissue can be detected [4]. Endometriomas affect approximately 17–44% of patients with endometriosis [5]. These cysts are most often unilateral, with a mean cyst wall thickness of 1.2–1.5 mm. When bilateral, endometriomas are associated with more extensive disease and posterior cul-de-sac obliteration. The left ovary is more commonly affected, potentially due to the anatomical position of the sigmoid colon, which allows endometrial fragments from the uterine cavity to persist and implant on the left ovary. Ovarian endometriomas may be associated with infertility, dysmenorrhea, chronic pelvic pain, ovarian masses, or bowel upset (i.e., swelling or fullness) [6].

##### Management, Treatment, and Clinical Outcomes

Evidence-based guidelines recommend medical therapy as the first-line approach for patients with endometrioma-related pain who do not desire pregnancy. Medical treatment can improve patients’ quality of life and reduce cyst size and generally poses a low level of risk [7]. Surgery is considered for patients with large endometriomas (e.g., size > 3 cm), those whose symptoms do not adequately improve or worsen, or those whose imaging results suggest malignancy. Medications that can reduce the size and symptoms of endometriomas include progestins, oral estrogen–progestin contraceptive pills, and gonadotropin-releasing hormone (GnRH) agonists or antagonists. The choice of medication is based on patient preferences, including considerations related to contraception use, side effects, drug availability, and cost. For example, in young women, oral contraceptives (OCs) are often preferred because they provide adequate estrogen support, which is essential for improving their quality of life [8]. It is advisable to use preparations with the lowest possible estrogen content to prevent endometrial proliferation [9]. Continuous-dose OC regimens may provide additional benefits over cyclic ones. A continuous OC schedule is in fact associated with lower dysmenorrhea and/or endometrioma recurrence rates compared with a cyclic schedule [10]. Progestin monotherapy (e.g., dienogest or norethindrone acetate (NETA)) is historically favored in women who do not respond to combined hormone therapy or as a first-line therapy in smokers over 35 years old, women with a history of migraines, and those with predisposing risk factors for thromboembolic events [11].

Patients with more severe symptoms or whose symptoms are not adequately controlled with the above treatments are offered a trial of GnRH antagonists [12]. GnRH antagonists (e.g., elagolix or relugolix) suppress pituitary gonadotropin hormone production and create a dose-dependent hypoestrogenic state to inhibit endometriotic cell proliferation. Compared to GnRH analogues, antagonists do not provoke the initial flare-up phase, and the onset of their therapeutic effect is rapid [13]. They also have the advantage of being administered orally. Symptom relief and adverse events, such as vasomotor phenomena, vaginal atrophy, and bone loss, are also dose-dependent. When used with add-back hormone therapy, adverse effects such as menopause-like symptoms and bone density decrease are reduced. According to Wang et al. [14], two recently FDA-approved doses of elagolix for the management of moderate-to-severe pain associated with endometriosis were both proven to be cost effective compared to leuprolide acetate (i.e., GnRh analogues) over a time frame of 1–2 years. However, studies comparing the efficacy of GnRH antagonists with OC and progestins are required.

For patients with infertility, additional considerations are necessary. These women can be managed with either surgery or in vitro fertilization (IVF) [15]. Currently, the decision between surgery and IVF is shared and tailored to the patient’s specific circumstances, including their history of previous surgeries, pain symptoms, age, ovarian reserve testing results, and semen analysis [16]. The lack of randomized trials comparing these two strategies makes precise estimations challenging. Surgery might increase the chances of natural conception and relieve symptoms [17,18]. IVF may be more effective but involves higher costs and specific risks during the IVF procedure and pregnancy for women with endometriosis who do not undergo surgery. The presence of the endometrioma may in fact interfere with ovarian responsiveness to hyperstimulation and oocyte competence, the retrieval of the oocytes may be more difficult and riskier, the disease may progress during the procedure, one’s pregnancy outcome may be affected, and there is a risk of missing occult malignancies with cancer development later in life [19]. An ongoing randomized controlled trial (RCT) by Ottolina et al. aims to provide clinically relevant findings in this context [20].

When performing surgery on women with ovarian endometriosis, clinicians may consider both cystectomy and CO_2_ laser vaporization, as both techniques have similar recurrence and reproductive rates [21,22]. In CO_2_ laser vaporization, the “pseudo-capsule” of the cyst is not removed but ablated with the CO_2_ laser. This laser is highly selective and precise, with a minimal depth of tissue penetration and little lateral thermal spread, reducing the risk of unintended thermal damage. Additionally, the CO_2_ laser allows for the continuous visualization of the section plane between healthy and endometriosis-affected tissue. These properties are crucial for preserving the surrounding viable ovarian tissue. The CO_2_ laser also simultaneously cauterizes bleeding tissue, providing effective hemostasis without the risks associated with cautery. The introduction of the CO_2_ fiber laser has made the treatment of endometriotic cysts feasible even for non-expert surgeons [23].

#### 3.1.2. Superficial Peritoneal Endometriosis

Superficial peritoneal endometriosis is characterized by the presence of ectopic endometrium-like tissue outside the uterus, extending up to 5 mm under the peritoneal pelvic surface and/or the serosa of the pelvic viscera [24]. The prevalence of this condition is difficult to assess due to the poor sensitivity of non-invasive diagnostic tools, such as transvaginal ultrasound [1]. Despite this, it is the most common subtype of endometriosis, present in up to 80% of women affected by the disease and representing the only clinical finding in 30% of laparoscopically confirmed endometriosis cases [25,26]. Clinically, superficial endometriosis can be asymptomatic in 40% of cases and it is found when women undergo laparoscopies for other clinical reasons [27]. However, it has been associated with primary infertility, moderate to severe dysmenorrhea, and deep dyspareunia [26]. Given these associations, superficial endometriosis should be suspected in women of reproductive age presenting with infertility and/or painful symptoms, even in the absence of diagnostic clinical findings, and empiric treatment should be initiated.

##### Management, Treatment, and Clinical Outcomes

For symptomatic women with suspected superficial endometriosis who do not desire pregnancy, the first-line treatment recommended by international guidelines is medical hormonal therapy. Options include combined hormonal contraceptives, progestins, GnRH agonists, or GnRH antagonists, all of which aim to suppress estrogen stimulation. The choice of treatment is based on their effectiveness, side effects, long-term safety, cost, and availability [1]. However, up to 30% of women do not achieve symptom relief with medical therapy, and some patients have contraindications to hormonal treatment [1,28]. In such cases, a laparoscopy should be considered.

Minimally invasive surgical techniques, such as excision and ablation through coagulation or CO_2_ laser vaporization, are available. Current guidelines recommend surgical excision over ablation to reduce endometriosis-associated pain and provide histological confirmation [1]. Recent randomized controlled trials (RCTs) have compared the effectiveness of laparoscopic excision and ablation for superficial endometriosis-related pain. One trial found a significant improvement in dyspareunia after ablation at 6 months, but this improvement was not sustained at 12 months, with excision and ablation showing similar levels of effectiveness overall for pain management [29,30,31].

Superficial endometriosis is also associated with primary infertility, though not necessarily due to a diminished ovarian reserve, as serum antimüllerian levels and antral follicle counts are comparable to those of women without endometriosis [32]. Their infertility may instead be due to factors such as distorted anatomy, inflammation, and epigenetic changes [33]. The management of infertility in women with superficial endometriosis remains debated. The European Society of Human Reproduction and Embryology (ESHRE) recommends surgical treatment for endometriosis-associated infertility in stage I/II endometriosis due to an improvement in the rate of spontaneous ongoing pregnancy, though an increase in live birth rates has not been demonstrated [1]. Assisted reproductive technologies (ART) can be considered before or after surgery if there are issues such as compromised tubal function, male factor infertility, or a poor prognosis [34]. Thus, decisions on managing infertility related to superficial endometriosis should be individualized, considering the presence of painful symptoms, the patient’s age, preferences, and available fertility treatment options [35].

#### 3.1.3. Deep Infiltrating Endometriosis

Deep infiltrating endometriosis (DIE) is the most severe form of endometriosis, characterized by the involvement of either retroperitoneal structures or the peritoneum to a depth of at least 5 mm [36]. It affects approximately 1% of women of reproductive age and up to 20% of women diagnosed with endometriosis [37]. Common locations include the torus uteri, posterior fornix, uterosacral ligaments, vagina, bowel, and urinary tract. DIE significantly reduces the chances of natural conception, likely due to a combination of anatomical distortion, dyspareunia, and pelvic inflammation.

##### Management, Treatment, and Clinical Outcomes

When creating a treatment plan for DIE, clinicians must consider the patient’s symptoms, age, desire for future pregnancy, location and type of lesions, and the risks and benefits of both medical and surgical therapies. Evidence-based guidelines recommend medical therapy as the first approach for patients with deep endometriosis-related pain who do not desire pregnancy [38,39]. Exceptions include patients with significant bowel or ureteral obstruction, who require immediate surgical intervention [40]. Medical treatment can substantially improve symptoms (approximately 70–80%) and avoid the risks associated with surgery, such as genital tract fistulae, bladder dysfunction, or bowel anastomotic leakage [41]. Patients eligible for hormonal treatment should be counseled about the potential need for surgery in case of treatment failure (approximately 10%) and the possible development of a bowel or ureteral obstruction (1–2%).

Both combined estrogen–progestin and progestin-only regimens are effective in reducing symptoms. Some authors suggest progestin-only regimens as a first-line treatment (e.g., norethindrone acetate [NETA] (2.5 mg) or dienogest (2 mg)) [42]. Additional medical treatment options include the short-term use of gonadotropin-releasing hormone agonists [40]. In cases of infertility-related endometriosis, clinicians must choose between surgical intervention and assisted reproductive technologies (ARTs).

The impact of surgery for DIE on fertility is controversial, with the literature presenting heterogeneous findings. A Brazilian prospective study comparing IVF outcomes in women who underwent extensive endometriosis excision before IVF (64 patients) and those who only underwent IVF (105 patients) found significantly higher implantation (32.1% vs. 19%) and pregnancy rates (41% vs. 24%) in the surgical group, despite higher r-FSH dosages and fewer oocytes being retrieved [43]. However, the study did not specify the types of endometriotic lesions in both groups. A retrospective observational study evaluated factors associated with pregnancy during the first two IVF attempts in infertile women with posterior deep endometriosis (lesions in the torus, uterosacral ligaments, recto-uterine space, rectovaginal septum, and/or bowels), finding a 48.7% pregnancy rate after two IVF attempts, with lower chances of pregnancy in the presence of a recto-uterine nodule [44,45].

Operative laparoscopy may be a treatment option for symptomatic patients wishing to conceive, though some patients may require surgery prior to ART to free the ovaries from adhesions, facilitating oocyte retrieval. Surgical decisions should be made by a multidisciplinary team with a high level of surgical expertise to minimize risks and maximize benefits. There is no consensus on the best surgical treatments for various forms of DIE. The critical point is to balance radicality and conservativity to improve patient quality of life while minimizing surgical complications [40]. Surgeons should identify, isolate, and remove all sites of endometriosis in the pelvis, following nerve-sparing principles, especially in posterior compartment DIE [46]. Sparing hypogastric nerves and inferior hypogastric plexuses is crucial to avoid autonomic nervous injury, which can cause urinary, anorectal, and sexual dysfunction [47].

Surgical techniques for bowel endometriosis include shaving, discoid excision, or segmental resection [48]. A recent meta-analysis found that shaving had the lowest postoperative complication rate but a higher incidence of recurrence compared to discoid excision and segmental resection [49,50,51]. An RCT (EndoRE trial) comparing conservative (shaving and disc excision) and radical (segmental bowel resection) approaches found high conception rates during follow-up (82.4% with radical and 85% with conservative approaches), with a 75.7% live birth rate and a 57.4% spontaneous pregnancy rate (SPR) [50,52]. Positive results were previously obtained by Bourdel et al., who reported a 49% SPR after shaving and 67% after resection, and by Donnez et al., who reported a 57% SPR after the shaving technique [53,54]. However, two prospective studies did not show significant differences [55,56]. An observational study by Stepniewska et al. found a cumulative pregnancy rate of 58% among women younger than 30 years and 45% in those aged 30–34 years following laparoscopic colorectal resection for severe intestinal symptoms in infertile women with endometriosis [57].

More recently, a retrospective matched cohort study concluded that first-line surgery may be a good option for women with colorectal endometriosis-associated infertility compared with IVF without surgery [58]. However, the groups were heterogeneous in terms of the surgery performed, including rectal shaving or segmental colorectal surgery based on infiltration extent, ovarian endometrioma cystectomy or ablation using plasma energy, salpingectomy, uterosacral ligament resection, partial colpectomy, partial bladder resection, ureterolysis, and ureteral reimplantation.

Regarding urinary tract endometriosis, surgical techniques depend on the type and location of lesions [40]. Ureterolysis is preferred for extrinsic ureteral endometriosis, while ureteral resection with end-to-end anastomosis is necessary for intrinsic ureteral endometriosis when conservative approaches fail. For lesions near the vesical–ureteral junction, ureteral reimplantation is considered. Surgery for bladder endometriosis involves a partial cystectomy, with or without the opening of the bladder cavity [59]. Bladder DIE often coexists with other locations, complicating assessments of its impact on infertility and surgical outcomes [60]. Retrospective studies have reported spontaneous pregnancy rates (SPR) ranging from 30% to 86%, with no significant differences between complete or partial thickness bladder DIE [61,62].

In summary, managing DIE involves careful consideration of various factors, with a multidisciplinary approach to optimize patient outcomes. Both medical and surgical treatments can be used and tailored to individual patient needs and circumstances.

### 3.2. Adenomyosis

Uterine adenomyosis involves the infiltration of endometrial glands and stroma into the myometrium, resulting in the hypertrophy of adjacent myometrial tissue [63]. This condition presents as a heterogeneous pathology, with diffuse, focal, and adenomyoma phenotypes within the myometrium.

The exact cause of adenomyosis remains unknown, but recent theories suggest the mechanism of tissue injury and repair (TIAR) and stem cell theory. The dysfunction or absence of the endomyometrial junctional zone (JZ) may lead to internal or intrinsic adenomyosis through the invagination of the basal layer of the endometrium into the myometrium [64]. However, an alternative theory suggests that adenomyotic lesions may develop from the metaplasia of misplaced embryonic pluripotent Mullerian remnants or indeed from the differentiation of adult stem cells [65].

Currently, adenomyosis diagnosis primarily relies on non-invasive imaging techniques, particularly pelvic ultrasound and pelvic MRI, both of which demonstrate high levels of sensitivity and specificity. In a meta-analysis involving over 3300 patients with confirmed adenomyosis, transvaginal ultrasound (TVUS) exhibited a sensitivity and specificity of 81% and 87%, respectively, while MRI showed a 71% sensitivity and 91% specificity [66].

A consensus-based practical classification of adenomyosis has been proposed by T. Van Den Bosch et al., based on ultrasound findings. This classification system includes the following:

1. Identification of the presence of adenomyosis using the MUSA criteria [67];
2. Determination of the location of the adenomyosis;
3. Differentiation between focal and diffuse disease;
4. Discrimination between cystic and non-cystic lesions;
5. Determination of myometrial layer involvement;
6. Classification of disease extent as mild, moderate, or severe;
7. Measurement of lesion size.

However, international consensus on adenomyosis classification is still lacking.

Moreover, studies have highlighted associations between sonographic features of adenomyosis and clinical presentation. Diffuse adenomyosis of the internal myometrium tends to occur more frequently in older, multiparous women, often associated with prior uterine surgery [68]. Conversely, focal adenomyosis of the external myometrium is more prevalent in younger women and is often linked to deep endometriosis [68]. The presence of abnormal uterine bleeding and infertility varies according to adenomyosis phenotype, with diffuse adenomyosis being more strongly associated with abnormal bleeding and external adenomyosis more prevalent in cases of infertility [69].

Assessing adenomyosis’s impact on reproductive outcomes is challenging due to the limited differentiation between endometriosis and adenomyosis in many studies. However, evidence suggests that adenomyosis may have a greater impact on the likelihood of conception and the risk of miscarriage than endometriosis alone. Additionally, women with adenomyosis face an increased risk of developing preeclampsia and delivering small-for-gestational-age (SGA) infants, likely due to the mechanisms of poor placentation and endothelial inflammation [70].

In summary, the diagnosis and classification of adenomyosis rely heavily on non-invasive imaging techniques, although international consensus on classification is lacking. Adenomyosis phenotype correlates with clinical presentation, and further research is needed to better understand its impact on reproductive outcomes.

#### Management, Treatment, and Clinical Outcome

The management of adenomyosis focuses on symptom alleviation, improving patients’ quality of life, and addressing associated complications (e.g., ureteral obstructions). Treatment choices depend on factors such as symptom severity, fertility desires, and overall health.

Hormonal therapies are commonly used to alleviate adenomyosis symptoms by suppressing menstruation, reducing endometrial tissue growth, and diminishing inflammatory cytokines and prostaglandins. The most effective first-line therapy for symptomatic patients not desiring pregnancy is the 52 mg levonorgestrel-releasing intrauterine device (LNG 52). It acts directly on the uterus, maintains low systemic hormone levels, and offers long-acting, user-independent administration [71]. Observational studies indicate its efficacy in improving heavy menstrual bleeding and dysmenorrhea associated with adenomyosis [72].

Other hormonal strategies, such as progestins, oral contraceptive pills, and gonadotropin-releasing hormone (GnRH) agonists and antagonists, are effective alternatives for managing heavy menstrual bleeding and dysmenorrhea in patients unable to use LNG 52. However, data on their efficacy are limited.

Adenomyomectomy may be considered for symptomatic patients with focal adenomyosis who desire fertility preservation. However, adenomyomas are challenging to excise due to difficulties in establishing a surgical plane and the woody consistency of the adenomyotic uterus. Additionally, suturing is difficult in this environment. Even if performed by expert surgeons, adenomyomectomy carries a risk of uterine rupture in future pregnancies, reported to be around 4% [73,74].

For patients who have completed childbearing or for whom medical therapies are ineffective or contraindicated, hysterectomies remain as a definitive treatment option [75]. A hysterectomy may be considered a valid option in cases of adenomyosis, especially when other treatments have failed to alleviate symptoms such as severe pain and heavy menstrual bleeding. However, its effectiveness in resolving pain is not guaranteed. The persistence of pain can result from residual endometriotic lesions, central sensitization, or hormonal influences if the ovaries are retained. Therefore, a comprehensive evaluation and an individualized treatment plan, often involving a combination of surgical and non-surgical approaches, are essential for optimal management.

Uterine artery embolization (UAE) is a non-operative alternative treatment for adenomyosis, aimed at reducing associated symptoms. Studies have shown a 25% decrease in uterine volume and improvement in abnormal bleeding and overall symptoms following UAE [76,77].

Acupuncture has been explored as a complementary therapy for managing adenomyosis symptoms. However, evidence regarding its effectiveness remains limited and inconclusive [78].

The goal of adenomyosis management is to alleviate symptoms, improve patient quality of life, and address complications while considering patient preferences and fertility desires. Treatment outcomes vary depending on the chosen approach: hormonal therapies offer symptom relief, adenomyomectomies preserve fertility, and hysterectomies are used when other treatments have failed to alleviate severe pain and heavy menstrual bleeding. Non-operative options like UAE and complementary therapies like acupuncture offer alternative symptom management strategies. Further research is needed to assess the long-term efficacy and outcomes of these treatments.

### 3.3. Alternative Approaches for Managing Endometriosis-Related Symptoms

Holistic, complementary, and alternative approaches for managing endometriosis often focus on relieving symptoms and improving overall well-being. Some approaches include the following:

1. Dietary Changes: Certain diets, such as an anti-inflammatory diet rich in fruits, vegetables, whole grains, and healthy fats, may help alleviate symptoms. Some people find relief by avoiding trigger foods like dairy or gluten.
2. Acupuncture: Acupuncture, a traditional Chinese medicine practice, involves inserting thin needles into specific points on the body to alleviate pain and promote relaxation. Some women with endometriosis find acupuncture helpful for managing pain and stress.
3. Herbal Supplements: Certain herbs like turmeric, ginger, and chasteberry (vitex) are believed to have anti-inflammatory properties and may help reduce pain associated with endometriosis. However, it is essential to consult with a healthcare provider before trying herbal supplements, as they can interact with medications or have side effects.
4. Mind–Body Practices: Techniques such as yoga, meditation, and mindfulness can help manage stress, improve sleep quality, and promote relaxation. These practices may help reduce pain and enhance overall well-being in individuals with endometriosis.
5. Physical Therapy: Pelvic floor physical therapy can help address pelvic pain and dysfunction associated with endometriosis. Therapists use techniques such as manual therapy, stretching, and strengthening exercises to alleviate pain and improve pelvic function.
6. Supplements: Some supplements, such as omega-3 fatty acids, magnesium, and vitamin D, may help reduce inflammation and alleviate symptoms of endometriosis. However, it is essential to consult with a healthcare provider before taking supplements to ensure they are safe and appropriate for one’s specific situation.
7. Stress Management: Chronic stress can exacerbate symptoms of endometriosis. Practices like deep breathing exercises, progressive muscle relaxation, and biofeedback can help manage stress levels and reduce the impact of stress on symptoms.

It is important to note that while these holistic approaches may provide symptom relief for some individuals, they should not replace conventional medical treatment.

## 4. Discussion

Endometriosis and adenomyosis present complex challenges in terms of their diagnosis, management, and treatment, often requiring a multidisciplinary approach. Despite extensive research, certain aspects of their management remain controversial, necessitating ongoing studies to refine treatment strategies and improve patient outcomes.

1. Need for Specialized Care: The complexity of endometriosis and adenomyosis necessitates referral to specialized facilities in which patients can benefit from specific high-quality management, from early diagnosis to the treatment of severe disease. Given the significant impact of endometriosis on women’s daily lives and its economic burden, gynecological societies and international organizations advocate for the establishment of expert centers throughout the country, formally accredited by health authorities, ideally as part of a National Health Plan [79].
2. Challenges in Managing Infertility: Infertility associated with endometriosis poses a significant challenge. The decision between surgery and in vitro fertilization (IVF) depends on various factors, including previous surgical history, pain symptoms, age, and ovarian reserve. Robust prospective studies, such as multicenter randomized controlled trials (RCTs), are essential to compare the effectiveness of these approaches and guide clinical decision making.
3. Ongoing Clinical Trials: The ongoing multicenter RCTs comparing IVF and surgery for ovarian endometriosis and deep infiltrating endometriosis (DIE) will provide valuable insights into their respective benefits and risks, particularly in terms of live birth rates [78]. Additionally, this research will also include an experimental part aimed at assessing whether the systemic inflammatory environment of endometriosis may have a detrimental impact on the quality of folliculogenesis and embryological development.
4. Future Directions in Adenomyosis Research: Adenomyosis poses unique challenges due to its heterogeneity and limited treatment options, especially in cases in which pregnancy is desired. The lack of RCTs comparing different treatment strategies highlights the need for further research to identify optimal management approaches. Studies investigating the role of gonadotropin-releasing hormone (GnRH) agonists in improving assisted reproductive technology (ART) outcomes in patients with severe adenomyosis offer promising insights. However, more research is needed to establish standardized protocols and evaluate their efficacy.
5. Obstetric Complications: Women with severe endometriosis and adenomyosis face increased risks of obstetric complications, including preeclampsia, antepartum hemorrhage, preterm birth, and miscarriage. Understanding these associations is crucial for optimizing prenatal care and reducing maternal and fetal risks.

## 5. Conclusions

Ongoing research efforts, including RCTs and multicenter cohort studies, are essential for advancing our understanding and management of endometriosis and adenomyosis. Collaborative initiatives involving multidisciplinary teams will facilitate the development of evidence-based guidelines and improve patient care outcomes.

## Data Availability

Not applicable.
